# Direct (Hetero)Arylation Polymerization of a Spirobifluorene and a Dithienyl-Diketopyrrolopyrrole Derivative: New Donor Polymers for Organic Solar Cells

**DOI:** 10.3390/molecules23040962

**Published:** 2018-04-20

**Authors:** Pierre Josse, Sergey Dayneko, Yangqian Zhang, Sylvie Dabos-Seignon, Shiming Zhang, Philippe Blanchard, Gregory C. Welch, Clément Cabanetos

**Affiliations:** 1 CNRS UMR 6200, MOLTECH-Anjou, University of Angers, 2 Bd Lavoisier, 49045 Angers, France; pierre.josse@univ-angers.fr (P.J.); sylvie.dabos@gmail.com (S.D.-S.); philippe.blanchard@univ-angers.fr (P.B.); 2 Department of Chemistry, University of Calgary, 2500 University Drive N.W., Calgary, AB T2N 1N4, Canada; sergey.dayneko@ucalgary.ca (S.D.); gregory.welch@ucalgary.ca (G.C.W.); 3 Key Laboratory of Flexible Electronics (KLOFE) & Institute of Advanced Materials (IAM), Jiangsu National Synergetic Innovation Center for Advanced Materials (SICAM), Nanjing Tech University, 30 South Puzhu Road, Nanjing 211816, China; iamyqzhang@njtech.edu.cn (Y.Z.); iamsmzhang@njtech.edu.cn (S.Z.)

**Keywords:** direct (hetero)arylation polymerization, spirobifluorene, diketopyrrolopyrrole, organic photovoltaics

## Abstract

The synthesis and preliminary evaluation as donor material for organic photovoltaics of the poly(diketopyrrolopyrrole-spirobifluorene) (**PDPPSBF**) is reported herein. Prepared via homogeneous and heterogeneous direct (hetero)arylation polymerization (DHAP), through the use of different catalytic systems, conjugated polymers with comparable molecular weights were obtained. The polymers exhibited strong optical absorption out to 700 nm as thin-films and had appropriate electronic energy levels for use as a donor with **PC_70_BM**. Bulk heterojunction solar cells were fabricated giving power conversion efficiencies above 4%. These results reveal the potential of such polymers prepared in only three steps from affordable and commercially available starting materials.

## 1. Introduction

Due to their potential low cost, light-weight, compatibility with large-surface processing techniques and low-environmental impact, organic photovoltaic cells (OPVs) have generated, in just few years, a general enthusiasm within the scientific community. As a result, power conversion efficiencies (PCEs) have been significantly improved now reaching 13% due to the recent advent of non-fullerene acceptors [1,2,3]. However, this race to efficiencies, although essential from an industrial point of view, appears to be in stark contrast with the main advantage of this technology which is to primarily afford inexpensive and cost-effective materials. Indeed, efficient active materials reported in the literature generally require numerous synthetic and purification steps that lower the overall yields while generating large amounts of chemical wastes. This has rendered many top performing materials expensive and not suitable for solar cell mass production [4,5,6,7]. Hence, to avoid this cryptic situation, the direct (hetero)arylation (DHA) appears to be method of choice, from the chemist point of view, to afford the next generation of π-conjugated materials [8,9,10,11,12,13,14,15]. The latter indeed offers several practical advantages such as (*i*) a simplified and shortened synthetic route; (*ii*) a better stability of the monomers; (*iii*) the prevention of metal-substituted terminal groups; (*iv*) a lower environmental impact, since organometallic transmetallation derivatives are no longer necessary and (*v*) the unique opportunity of affording new compounds that cannot be achieved with the traditional methods.

In a further expression of our commitment toward simplified structures for organic photovoltaics [16,17,18,19,20,21,22,23], we report herein the synthesis of a poly(diketopyrrolopyrrole-spirobifluorene) (**PDPPSBF**) polymer via homogeneous and heterogeneous direct (hetero)arylation polymerization (DHAP) for OPV applications. Prepared in a maximum of three steps from affordable and commercially available materials, this macromolecular donor was built around a design principle where a bulky electron-rich monomer is copolymerized with a more sterically accessible electron-accepting moiety to maximize the interaction with the fullerene derivatives [24].

## 2. Results and Discussion

**Scheme 1:**
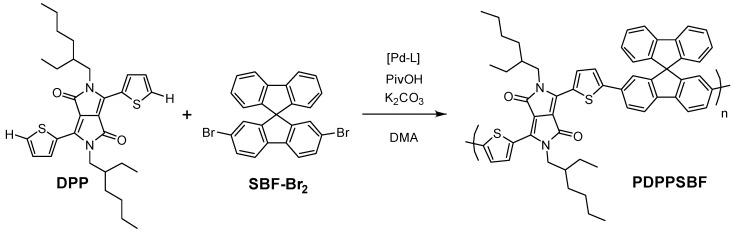
Synthesis of the poly(diketopyrrolopyrrole-spirobifluorene) (**PDPPSBF**) polymer.

Widely involved in efficient active materials, the synthetically accessible dithiophenyldiketopyrrolopyrrole (**DPP**), prepared in only two steps and in gram scale [25], was co-polymerized with the commercially available 2,7-dibromo-9,9′-spirobi[fluorene] (**SBF-Br_2_**) via direct (hetero)arylation (Scheme 1).

To achieve this goal, three different catalytic systems, involved in the functionalization of **DPP** derivatives via DHA, were used and evaluated herein [25,26,27,28]. However, for comparative purposes, all polymerizations were all carried out in dimetylacetamide at 80 °C in presence of potassium carbonate and pivalic acid.

For both homogeneous systems (**P1** and **P2**, Table 1), the color of the reaction quickly changed from dark pink to violet then greenish. After 24 h, the polymer mixtures were precipitated into methanol, purified by Soxhlet extraction, and isolated as dark solids. Gel permeation chromatography (GPC) analyses were then performed (Figures S5–S7) and revealed, in consistency with the polymerization yields, a significant difference in the number average molecular weight (M*n*) and molar-mass dispersity (Đ_m_). Longer chain polymers were obtained using the palladium acetate-based catalyst system while only short oligomers were obtained using the Herrmann–Beller catalyst system. In parallel, first attempts of polymerization using the heterogeneous and commercially available Silia*Cat*^®^ DPP-Pd [29] in similar reacting conditions, i.e., 5 mol % of catalyst, systematically led to a mixture of starting materials and traces of soluble short oligomers (**P3**). To overcome this problem, the concentration of supported palladium was doubled, leading to polymer **P4** with comparable M*n* than those obtained in homogenous conditions with the palladium acetate/tricyclohexylphosphine tetrafluoroborate system.

**Figure 1:**
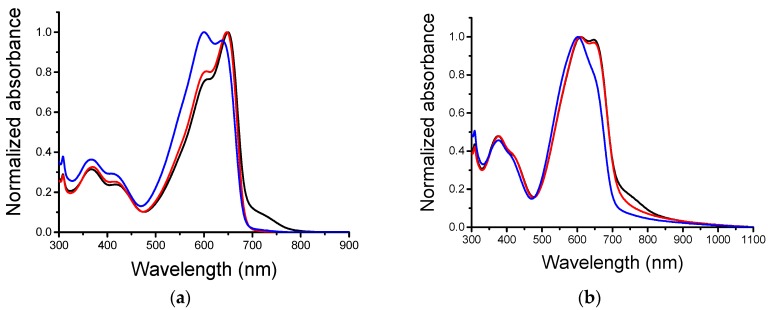
Normalized UV-Vis absorption spectra of the **P1** (red) **P2** (blue) and **P4** (black) in solution (**a**) and as thin film on glass (**b**).

Soluble in chloroform, the three isolated polymers, namely **P1**, **P2** and **P4,** were characterized by UV-visible spectroscopy revealing strong optical absorption in the visible region (Figure 1).

Two different optical absorption profiles were recorded and correlated to the molecular weights. The shorter chain polymer **P2** exhibits a well-resolved vibrational structure with two equivalent maxima at ca. 638 nm and 599 nm. For the longer chain polymers **P1** and **P4** the intensity of the most energetic band decreases with the lowest energy peak becoming more prominent which is consistent with aggregation of the polymer structure. In addition, further investigation of the spectra of **P4** also reveals extra transitions beyond 700 nm attributed to **DPP** homo-coupling defects along the chains [14,30,31,32]. We hypothesize that this issue might be attributed to the sluggish and step-wise nature of catalytic cycle using the Silia*Cat*^®^ DPP-Pd catalyst, which starts as Pd(II) but has a Pd(0) resting state. Considering this is the first report of the use of this catalyst for the synthesis of conjugated polymers further investigation is warranted. Conversely, the homo-coupling defect has been recently and properly harnessed by Welch et al. to prepare dimeric DPP-based molecular systems [33].

Upon transitioning from solution to the thin-film, slight shifts toward longer wavelengths region were observed in the optical absorption spectra of the three polymers indicating minor solid state packing effects, partly attributed to the steric hindrance generated by the spirobifluorene co-monomer (Table 2).

Thin films, prepared by drop casting the latter solutions on a platinum working electrode, were then characterized by cyclic voltammetry (Figure S8 in the Supporting Information). As expected, increasing the molecular weights and therefore the conjugation length and intermolecular interactions results in reducing the HOMO-LUMO band gap (Table 2). Moreover, and in agreement with the literature [30,35,36,37], comparison of **P1** and **P4** reveals that DPP-homo coupling defects, monitored in the UV-visible spectra, also contributes in reducing the band gap through a concomitant stabilization of the LUMO and a destabilization of the HOMO level. 

**Figure 2:**
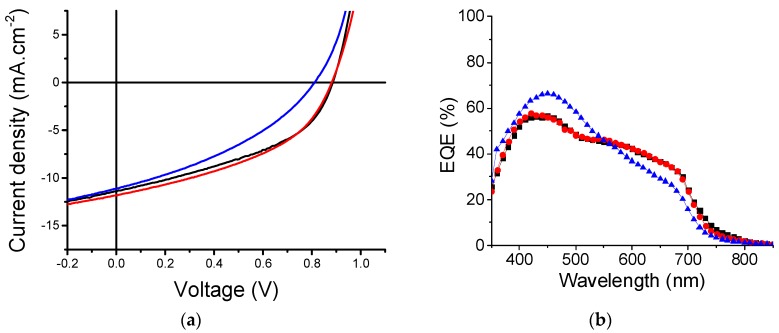
*J-V* characteristics (**a**) and the corresponding external quantum efficiencies (EQE) spectra (**b**) of the best solar cells from **P1**, **P2** or **P4** blended with **PC_70_BM**.

To evaluate their potential as donor materials and the impact of the polymerization method on the photovoltaic properties, simple air processed bulk heterojunction (BHJ) solar cells were, thereafter, fabricated. Hence, **P1**, **P2** and **P4** were combined with the [6,6]-phenyl-C_70_-butyric acid methyl ester (**PC_70_BM**) and embedded between a transparent ITO/PEDOT:PSS front contact and a reflective LiF/aluminum back contact electrode. Best devices were achieved from blends spun-cast at 1000 rpm from chloroform solutions in a 1:3 weight-to-weight donor:acceptor ratio at a concentration of 10 mg/mL. The corresponding density-voltage (*J-V*) curves and photovoltaic parameters are depicted and gathered in Figure 2 and Table 3 respectively.

It turns out that the shortest polymer (**P2**) gave the lowest power conversion efficiencies mainly impacted by lower open circuit voltages (V_oc_) and fill factor (FF) values. However, and interestingly, polymers of comparable length, namely **P1** and **P4** were characterized by comparable photovoltaic parameters, and therefore PCEs, despite the different nature of the catalytic systems used to prepare each material, i.e., homogeneous vs. heterogeneous.

This behaviors was particularly highlighted in the external quantum efficiencies (EQE) spectra since both responses are almost superimposable. Moreover, it also appears that the possible **DPP** homo coupling defects, emphasized in **P4** in the long wavelengths, have negligible effects on the photovoltaic characteristics [30,38]. On the other hand, although similar short circuit currents (J_sc_) of ca. 11 mA·cm^−2^ were recorded in the three cases, a different spectral response was monitored for **P2** resulting from an improved contribution in the acceptor region (400–500 nm) that compensates the lower photo conversion assigned to the polymer (550–700 nm).

**Figure 3:**
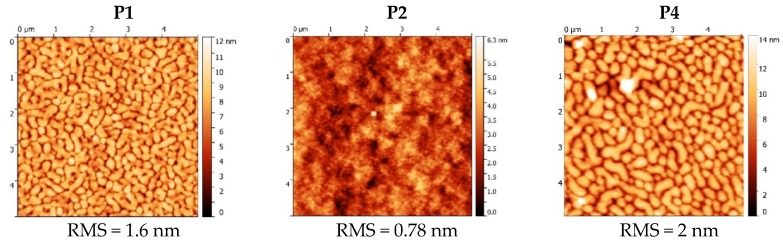
Atomic force microscopy (AFM) surface topography images of the **P1**, **P2** and **P4** based optimized active layers (RMS = Root Mean Square).

Consequently, to further rationalize these results the nanoscale topographies of the optimized blends were finally investigated by atomic force microscopy (Figure 3 and Figure S9).

The higher molecular weight polymers **P1** and **P4** based active layers exhibit comparable morphologies, characterized by well-organized and segregated micro domains. The blend made from the lower molecular weight polymer **P2** shows, *a contrario*, a smoother and homogeneous surface with coarse and nanometric structures. Here the higher molecular weight polymers promote phase-separation likely through polymer-polymer aggregation which is beneficial for achieving high photovoltaic performance These images once again highlight the key role of the morphology and its impact on the resulting photovoltaic characteristics [39,40,41,42].

## 3. Materials and Methods

All reagents and chemicals from commercial sources were used without further purification. Reactions were carried out under nitrogen atmosphere unless otherwise stated. Solvents were dried and purified using standard techniques.

### 3.1. Measurements and Characterization

Flash chromatography was performed with analytical-grade solvents using Aldrich (Saint Louis, MO, USA) silica gel (technical grade, pore size 60 Å, 230–400 mesh particle size). Flexible plates ALUGRAM^®^ Xtra SIL G UV254 from MACHEREY-NAGEL were used for TLC. Compounds were detected by UV irradiation (Thermo Fisher Scientific, Waltham, MA, USA) or staining with I_2_, unless stated otherwise. NMR spectra were recorded with a Bruker AVANCE III 300 (^1^H, 300 MHz and ^13^C, 75 MHz) (Bruker, Billerica, MA, USA). Chemical shifts are given in ppm relative to TMS and coupling constants (*J*) in Hz. Gel permeation chromatography (GPC) was conducted on a PL-GPC 220 instrument (Agilent, Santa Clara, CA, USA) with dichlorobenzene (DCB) as eluent against polystyrene standards. UV-vis spectra were recorded on a Shimadzu UV-1800 spectrometer (Shimadzu, Kyoto, Japan). Matrix Assisted Laser Desorption/Ionization was performed on MALDI-TOF MS BIFLEX III Bruker Daltonics spectrometer (Bruker Daltonics, Billerica, MA, USA) using dithranol as matrix. Cyclic voltammetry was performed using a Bio-Logic SP-150 potentiostat (Bio-Logic, Seyssinet-Pariset, France) with positive feedback compensation in 0.10 M Bu_4_NPF_6_/acetonitrile (HPLC grade, Thermo Fisher Scientific, Waltham, MA, USA). Experiments were carried out in a one-compartment cell equipped with a platinum working electrode (2 mm of diameter) and a platinum wire counter electrode. A silver wire immersed in 0.10 M Bu_4_NPF_6_/acetonitrile was used as pseudo-reference electrode and checked against the ferrocene/ferrocenium couple (Fc/Fc^+^) before and after each experiment. Atomic force microscopy (AFM) experiments were performed using the Nano-Observer device (CSInstrument, Les Ulis, France). The topographic images were obtained at room temperature in tapping mode. Images were processed with the Gwyddion free SPM data analysis software (v2.50, Czech Metrology Institute, Brno, Czech Republic).

### 3.2. Synthetic Procedures

2,5-bis(2-ethylhexyl)-3,6-di(thiophen-2-yl)pyrrolo[3,4-c]pyrrole 1,4(2*H*,5*H*)-dione **DPP** was synthetized according to previously reported method [25].

### 3.3. Polymerization of **PDPPSBF** in Homogeneous Conditions

**DPP** (50 mg; 1 eq), 2,7-dibromo-9,9′-spirobi[fluorene] (45 mg; 1 eq), potassium carbonate (33 mg; 2.5 eq), pivalic acid (3 mg; 0.3 eq), Palladium (Pd) catalyst (0.05 eq) and its ligand (L) (0.1 eq) were placed in a dry Schlenk tube equipped with a stir bar and degassed under vacuum. Dry and degassed dimethylacetamide (5 mL) was added to the powders and the reaction mixture was stirred at 80 °C under inert atmosphere for 24 h. After cooling down to room temperature the reaction mixture was precipitated in methanol before being filtered through a Soxhlet thimble and purified via Soxhlet extraction with methanol and hexanes successively. The polymer was finally extracted with chloroform. The resulting solution was then concentrated by evaporation, precipitated into methanol and filtered to afford the desired polymer.

**P1** (88%): Pd/L = Palladium(II) acetate/Tricyclohexylphosphine tetrafluoroborate; M*n* = 11,800 Da, PDI = 2.9; ^1^H NMR (300 MHz, CDCl_3_) δ = 8.86 (d, *J* = 3.6 Hz, 0.13H), 8.79 (d, *J* = 3.8 Hz, 1H), 7.90 (t, *J* = 7.8 Hz, 2H), 7.70 (d, *J* = 6.8 Hz, 1H), 7.60 (d, *J* = 4.8 Hz, 0.18H), 7.43 (t, *J* = 7.1 Hz, 1H), 7.15 (t, *J* = 7.3 Hz, 2H), 6.97 (s, 1H), 6.81 (d, *J* = 7.3 Hz, 1H), 3.96 (s, 2H), 1.80 (s, 2H), 1.27 (dd, *J* = 19.0, 9.9 Hz, 12H), 0.90–0.74 (m, 8H).

**P2** (49%): Pd/L = trans-bis(acetato)bis[*o*-(di-*o*-tolylphosphino)benzyl]dipalladium(II)/tris(*o*-methoxyphenyl)phosphine; M*n* = 2800 Da, PDI = 1.5; ^1^H NMR (300 MHz, CDCl_3_) δ = 8.86 (d, *J* = 3.4 Hz, 0.31H), 8.82–8.75 (m, 1H), 7.89 (dd, *J* = 11.1, 5.2 Hz, 2H), 7.71 (d, *J* = 6.3 Hz, 1H), 7.60 (d, *J* = 5.0 Hz, 0.36H), 7.43 (t, *J* = 7.5 Hz, 1H), 7.14 (q, *J* = 7.7 Hz, 1H), 6.97 (s, 1H), 6.81 (d, *J* = 7.7 Hz, 1H), 3.98 (s, 3H), 1.80 (s, 2H), 1.38–1.11 (m, 13H), 0.90–0.73 (m, 9H).

### 3.4. Polymerization of **PDPPSBF** in Heterogeneous Conditions

**DPP** (50 mg; 1 eq), 2,7-dibromo-9,9′-spirobi[fluorene] (45 mg; 1 eq), potassium carbonate (33 mg; 2.5 eq), pivalic acid (3 mg; 0.3 eq) and Silia*Cat* DPP-Pd^®^ (0.05 eq for **P3** and 0.1 eq for **P4**) were placed in a dry Schlenk tube equipped with a stir bar and degassed under vacuum. Dry and degassed dimethylacetamide (5 mL) was added to the powders and the reaction mixture was stirred at 80 °C under inert atmosphere for 24 h. After cooling down to room temperature the reaction mixture was precipitated in methanol before being filtered through a Soxhlet thimble and purified via Soxhlet extraction with methanol and hexanes successively. The polymer was finally extracted with chloroform. The resulting solution was then concentrated by evaporation, precipitated into methanol and filtered to afford the desired polymer.

**P3**: no precipitation in methanol.

**P4** (88%): M*n* = 10,900 Da, PDI = 2.3; ^1^H NMR (300 MHz, CDCl_3_) δ = 8.86 (d, *J* = 3.2 Hz, 0.16H), 8.79 (d, *J* = 3.9 Hz, 1H), 7.90 (t, *J* = 7.9 Hz, 2H), 7.70 (d, *J* = 7.6 Hz, 1H), 7.60 (d, *J* = 5.5 Hz, 0.19H), 7.42 (t, *J* = 7.4 Hz, 2H), 7.15 (t, *J* = 7.3 Hz, 2H), 6.97 (s, 1H), 6.81 (d, *J* = 7.5 Hz, 1H), 3.97 (s, 2H), 1.80 (s, 2H), 1.39–1.11 (m, 14H), 0.88–0.75 (m, 10H).

### 3.5. Device Fabrication and Testing

Pre-patterned indium-tin oxide coated glass slides of 24 × 25 × 1.1 mm with a sheet resistance of RS = 7 Ω/sq were purchased from Visiontek Systems. The substrates were washed by successive ultrasonic baths, namely diluted Deconex^®^ 12 PA-x solution (2% in water), acetone and isopropanol for 15 min each. Once dried under a steam of air, a UV-ozone plasma treatment (Ossila UV/Ozone cleaner E511) (Ossila, Sheffield, UK) was performed for 15 min. A filtered aqueous solution of poly(3,4-ethylenedioxy-thiophene)-poly(styrenesulfonate) (PEDOT:PSS; Ossila Al 4083) through a 0.45 μm RC membrane (Minisart^®^ RC 15) was spun-cast onto the ITO surface at 5000 rpm for 40 s before being baked at 120 °C for 30 min. Then, blends of **P1**, **P2** or **P4** and **PC_71_BM** (1:3 *w*/*w*) were dissolved in chloroform (10 mg/mL), stirred at 35 °C for 2 h and spun-cast onto the PEDOT:PSS layer at 1000 rpm for 30 s. Finally, devices were completed by the thermal deposition of lithium fluoride (0.5 nm) and aluminum (100 nm) at a pressure of 1.5 × 10^−5^ Torr through a shadow mask defining six cells of 27 mm^2^ each (13.5 mm × 2 mm). *J*-*V* curves were recorded in the dark and under illumination using a Keithley 236 source-measure unit and a home-made acquisition program. The light source is an AM1.5 Solar Constant 575 PV simulator (Steuernagel Lichttecknik, equipped with a metal halogen lamp, 100 mW·cm^−2^). The light intensity was measured by a broad-band power meter (13PEM001, Melles Griot). EQE were performed under ambient atmosphere using a halogen lamp (Osram) with an Action Spectra Pro 150 monochromator, a lock-in amplifier (Perkin-Elmer 7225) and a S2281 photodiode (Hamamatsu).

## 4. Conclusions

In summary and to move in directions that will improve on the eco-friendly and cost-efficient creed of the OPV community, we explored herein the synthesis, via direct (hetero)arylation polymerization, and preliminary evaluation of the simple and accessible **PDPPSBF** polymer as donor material in organic solar cells. Different catalytic systems were assessed and polymers with comparable molecular weights were obtained from both homogenous and heterogeneous conditions. Comparison of their electronic and electrochemical properties reveal similarities with the difference being that **DPP** homo-coupling defects were emphasized on the polymer prepared via the supported catalyst. However, the latter does not seem to affect their photovoltaic properties since similar power conversion efficiencies of ca 4.4% and morphologies were demonstrated. Hence, prepared in only three steps from commercially available and affordable building blocks, these polymers highlight, once again, the true potential of the direct (hetero)arylation as an efficient tool to design and afford simple, accessible and promising materials.

## Figures and Tables

**Table 1:** Number-average molecular weight (M*n*) and molar-mass dispersity (Đ_m_) after chloroform Soxhlet extraction according to the corresponding catalytic system.

Polymer PDPPSBF	Catalyst (mol %)	Ligand (mol %)	Yield (%)	M*n* (g·mol^−1^)	Đ_m_
**P1**	Pd(OAc)_2_ (5)	PCy_3_•HBF_4_ (10)	88	11,800	2.9
**P2**	Herrmann–Beller (5)	P(*o*-OMePh)_3_ (10)	49	2800	1.5
**P3**	Silia*Cat^®^* DPP-Pd (5)	---	---	---	---
**P4**	Silia*Cat^®^* DPP-Pd (10)	---	88	10,900	2.3

**Table 2:** Optical and electrochemical data. Potentials are expressed vs. Fc/Fc^+^ (E_HOMO_ = −(4.80 + E_ox_onset_); E_LUMO_ = −(4.80 + E_red_onset_)) [34].

Polymer PDPPSBF	λ_max_ (nm) in CHCl_3_	λ_max_ (nm) Film	λ_onset_ (nm) Film	E_red_onset_ (V)	E_ox_onset_ (V)	E_LUMO_ (eV)	E_HOMO_ (eV)
**P1**	648; 601; 416; 370	649; 608; 422; 376	720	−1.45	0.33	−3.35	−5.13
**P2**	638; 599; 412; 366	652; 601; 412; 377	700	−1.36	0.63	−3.44	−5.43
**P4**	649; 605; 418; 367	650; 608; 422; 375	813	−1.44	0.28	−3.36	−5.08

**Table 3:** Photovoltaic properties of **P1**, **P2** and **P4** blended with **PC_70_BM** and measured under an AM. 1.5 simulated solar illumination (100 mW·cm^−2^). Average value recorded over 14 devices; maximum values in brackets.

Polymer PDPPSBF	V_oc_ (V)	J_sc_ (mA·cm^−2^)	FF (%)	PCE (%)
**P1**	0.87 ± 0.03 (0.88)	11.47 ± 0.64 (11.84)	41 ± 1 (43)	4.10 ± 0.28 (4.47)
**P2**	0.80 ± 0.02 (0.81)	11.04 ± 0.77 (11.14)	32 ± 3 (36)	2.87 ± 0.33 (3.27)
**P4**	0.87 ± 0.03 (0.89)	11.51 ± 0.65 (11.42)	41 ± 2 (43)	4.13 ± 0.35 (4.31)

## Supplementary Materials

The following are available online. ^1^H NMR spectra (Figures S1–S4), cyclic voltammograms (Figure S5) and AFM images (Figure S6).

## References

[1] Zhao W., Li S., Yao H., Zhang S., Zhang Y., Yang B., Hou J. (2017). Molecular Optimization Enables over 13% Efficiency in Organic Solar Cells. J. Am. Chem. Soc..

[2] Hou J., Inganäs O., Friend R.H., Gao F. (2018). Organic solar cells based on non-fullerene acceptors. Nat. Mater..

[3] Yan C., Barlow S., Wang Z., Yan H., Jen A.K.Y., Marder S.R., Zhan X. (2018). Non-fullerene acceptors for organic solar cells. Nat. Rev. Mater..

[4] Po R., Bianchi G., Carbonera C., Pellegrino A. (2015). “All That Glisters Is Not Gold”: An Analysis of the Synthetic Complexity of Efficient Polymer Donors for Polymer Solar Cells. Macromolecules.

[5] Kirchartz T., Kaienburg P., Baran D. (2018). Figures of Merit Guiding Research on Organic Solar Cells. J. Phys. Chem. C.

[6] Min J., Luponosov Y.N., Cui C., Kan B., Chen H., Wan X., Chen Y., Ponomarenko S.A., Li Y., Brabec C.J. (2017). Evaluation of Electron Donor Materials for Solution-Processed Organic Solar Cells via a Novel Figure of Merit. Adv. Energy Mater..

[7] Po R., Roncali J. (2016). Beyond efficiency: Scalability of molecular donor materials for organic photovoltaics. J. Mater. Chem. C.

[8] Nitti A., Po R., Bianchi G., Pasini D. (2017). Direct Arylation Strategies in the Synthesis of π-Extended Monomers for Organic Polymeric Solar Cells. Molecules.

[9] Yu S., Liu F., Yu J., Zhang S., Cabanetos C., Gao Y., Huang W. (2017). Eco-friendly direct (hetero)-arylation polymerization: Scope and limitation. J. Mater. Chem. C.

[10] Bura T., Blaskovits J.T., Leclerc M. (2016). Direct (Hetero)arylation Polymerization: Trends and Perspectives. J. Am. Chem. Soc..

[11] Grenier F., Goudreau K., Leclerc M. (2017). Robust Direct (Hetero)arylation Polymerization in Biphasic Conditions. J. Am. Chem. Soc..

[12] Mercier L.G., Leclerc M. (2013). Direct (Hetero)Arylation: A New Tool for Polymer Chemists. Acc. Chem. Res..

[13] Pouliot J.-R., Grenier F., Blaskovits J.T., Beaupré S., Leclerc M. (2016). Direct (Hetero)arylation Polymerization: Simplicity for Conjugated Polymer Synthesis. Chem. Rev..

[14] Bura T., Beaupre S., Legare M.-A., Quinn J., Rochette E., Blaskovits J.T., Fontaine F.-G., Pron A., Li Y., Leclerc M. (2017). Direct heteroarylation polymerization: Guidelines for defect-free conjugated polymers. Chem. Sci..

[15] Liu F., Zhang Y., Wang H., Zhang S. (2018). Novel Conjugated Polymers Prepared by Direct (Hetero) arylation: An Eco-Friendly Tool for Organic Electronics. Molecules.

[16] Roncali J., Leriche P., Blanchard P. (2014). Molecular Materials for Organic Photovoltaics: Small is Beautiful. Adv. Mater..

[17] Labrunie A., Jiang Y., Baert F., Leliege A., Roncali J., Cabanetos C., Blanchard P. (2015). Small molecular push-pull donors for organic photovoltaics: Effect of the heterocyclic [small pi]-spacer. RSC Adv..

[18] Jiang Y., Cabanetos C., Allain M., Liu P., Roncali J. (2015). Manipulation of the band gap and efficiency of a minimalist push-pull molecular donor for organic solar cells. J. Mater. Chem. C.

[19] Grolleau J., Gohier F., Allain M., Legoupy S., Cabanetos C., Frère P. (2017). Rapid and green synthesis of complementary D-A small molecules for organic photovoltaics. Org. Electron..

[20] McAfee S.M., Dayneko S.V., Josse P., Blanchard P., Cabanetos C., Welch G.C. (2017). Simply Complex: The Efficient Synthesis of an Intricate Molecular Acceptor for High-Performance Air-Processed and Air-Tested Fullerene-Free Organic Solar Cells. Chem. Mater..

[21] Josse P., Dalinot C., Jiang Y., Dabos-Seignon S., Roncali J., Blanchard P., Cabanetos C. (2016). Phthalimide end-capped thienoisoindigo and diketopyrrolopyrrole as non-fullerene molecular acceptors for organic solar cells. J. Mater. Chem. A.

[22] Josse P., Labrunie A., Dalinot C., McAfee S.M., Dabos-Seignon S., Roncali J., Welch G.C., Blanchard P., Cabanetos C. (2016). Effect of side chains on the electronic and photovoltaic properties of diketopyrrolopyrrole-based molecular acceptors. Org. Electron..

[23] Labrunie A., Josse P., Dabos-Seignon S., Blanchard P., Cabanetos C. (2017). Pentaerythritol based push-pull tetramers for organic photovoltaics. Sustain. Energy Fuels.

[24] Graham K.R., Cabanetos C., Jahnke J.P., Idso M.N., El Labban A., Ngongang Ndjawa G.O., Heumueller T., Vandewal K., Salleo A., Chmelka B.F. (2014). Importance of the Donor:Fullerene Intermolecular Arrangement for High-Efficiency Organic Photovoltaics. J. Am. Chem. Soc..

[25] Hendsbee A.D., Sun J.-P., Rutledge L.R., Hill I.G., Welch G.C. (2014). Electron deficient diketopyrrolopyrrole dyes for organic electronics: Synthesis by direct arylation, optoelectronic characterization, and charge carrier mobility. J. Mater. Chem. A.

[26] Guo Q., Dong J., Wan D., Wu D., You J. (2013). Modular Establishment of a Diketopyrrolopyrrole-Based Polymer Library via Pd-Catalyzed Direct C–H (Hetero)arylation: A Highly Efficient Approach to Discover Low-Bandgap Polymers. Macromol. Rapid Commun..

[27] Gao Y., Zhang X., Tian H., Zhang J., Yan D., Geng Y., Wang F. (2015). High Mobility Ambipolar Diketopyrrolopyrrole-Based Conjugated Polymer Synthesized Via Direct Arylation Polycondensation. Adv. Mater..

[28] Areephong J., Hendsbee A.D., Welch G.C. (2015). Facile synthesis of unsymmetrical and [small pi]-extended furan-diketopyrrolopyrrole derivatives through C-H direct (hetero)arylation using a heterogeneous catalyst system. New J. Chem..

[29] Lemay M., Pandarus V., Simard M., Marion O., Tremblay L., Béland F. (2010). Silia*Cat*^®^ S-Pd and Silia*Cat* DPP-Pd: Highly Reactive and Reusable Heterogeneous Silica-Based Palladium Catalysts. Top. Catal..

[30] Hendriks K.H., Li W., Heintges G.H.L., van Pruissen G.W.P., Wienk M.M., Janssen R.A.J. (2014). Homocoupling Defects in Diketopyrrolopyrrole-Based Copolymers and Their Effect on Photovoltaic Performance. J. Am. Chem. Soc..

[31] Bohra H., Wang M. (2017). Direct C-H arylation: A “Greener” approach towards facile synthesis of organic semiconducting molecules and polymers. J. Mater. Chem. A.

[32] Lombeck F., Komber H., Gorelsky S.I., Sommer M. (2014). Identifying Homocouplings as Critical Side Reactions in Direct Arylation Polycondensation. ACS Macro Lett..

[33] Breukelaar W.B., McAfee S.M., Welch G.C. (2018). Exploiting direct heteroarylation polymerization homocoupling defects for the synthesis of a molecular dimer. New J. Chem..

[34] Cardona C.M., Li W., Kaifer A.E., Stockdale D., Bazan G.C. (2011). Electrochemical Considerations for Determining Absolute Frontier Orbital Energy Levels of Conjugated Polymers for Solar Cell Applications. Adv. Mater..

[35] Hayashi S., Yamamoto S.-I., Koizumi T. (2017). Effects of molecular weight on the optical and electrochemical properties of EDOT-based π-conjugated polymers. Sci. Rep..

[36] Botelho A.L., Shin Y., Liu J., Lin X. (2014). Structure and Optical Bandgap Relationship of π-Conjugated Systems. PLoS ONE.

[37] Roncali J. (2007). Molecular Engineering of the Band Gap of π-Conjugated Systems: Facing Technological Applications. Macromol. Rapid Commun..

[38] Lombeck F., Komber H., Fazzi D., Nava D., Kuhlmann J., Stegerer D., Strassel K., Brandt J., Mendaza A.D.D.Z., Müller C. (2016). On the Effect of Prevalent Carbazole Homocoupling Defects on the Photovoltaic Performance of PCDTBT:PC71BM Solar Cells. Adv. Energy Mater..

[39] Elumalai N.K., Uddin A. (2016). Open circuit voltage of organic solar cells: An in-depth review. Energy Environ. Sci..

[40] Hörmann U., Lorch C., Hinderhofer A., Gerlach A., Gruber M., Kraus J., Sykora B., Grob S., Linderl T., Wilke A. (2014). Voc from a Morphology Point of View: The Influence of Molecular Orientation on the Open Circuit Voltage of Organic Planar Heterojunction Solar Cells. J. Phys. Chem. C.

[41] Perez M.D., Borek C., Forrest S.R., Thompson M.E. (2009). Molecular and Morphological Influences on the Open Circuit Voltages of Organic Photovoltaic Devices. J. Am. Chem. Soc..

[42] Ray B., Lundstrom M.S., Alam M.A. (2012). Can morphology tailoring improve the open circuit voltage of organic solar cells?. Appl. Phys. Lett..

