# Supplementary material for: Direct (Hetero)Arylation Polymerization of a Spirobifluorene and a Dithienyl-Diketopyrrolopyrrole Derivative: New Donor Polymers for Organic Solar Cells
Source: Molecules. 2018 Apr 20;23(4):962. doi: 10.3390/molecules23040962 (PMC6017812; doi:10.3390/molecules23040962)
Supplement: Supplementary file 1 [file molecules-23-00962-s001.pdf]

SUPPORTING INFORMATIONS

Direct (Hetero)Arylation Polymerization of a Spirobifluorene and a Dithienyl-Diketopyrrolopyrrole Derivative: New Donor Polymers for Organic Solar Cells

Pierre Josse,[1] Sergey Dayneko,[2] Sylvie Dabos-Seignon, [1] Shiming Zhang,[3] Philippe Blanchard,[1] Gregory C. Welch,[2] Clément Cabanetos[1*]

[1]CNRS UMR 6200, MOLTECH-Anjou, University of Angers, 2 Bd Lavoisier, 49045 Angers, France

[2]Department of Chemistry, University of Calgary, 2500 University Drive N.W., Calgary, Alberta T2N 1N4, Canada

[3]Key Laboratory of Flexible Electronics (KLOFE) & Institute of Advanced Materials (IAM), Jiangsu National Synergetic Innovation Center for Advanced Materials (SICAM), Nanjing Tech University, 30 South Puzhu Road, Nanjing 211816, Jiangsu, P. R. China

Corresponding author: clement.cabanetos@univ-angers.fr

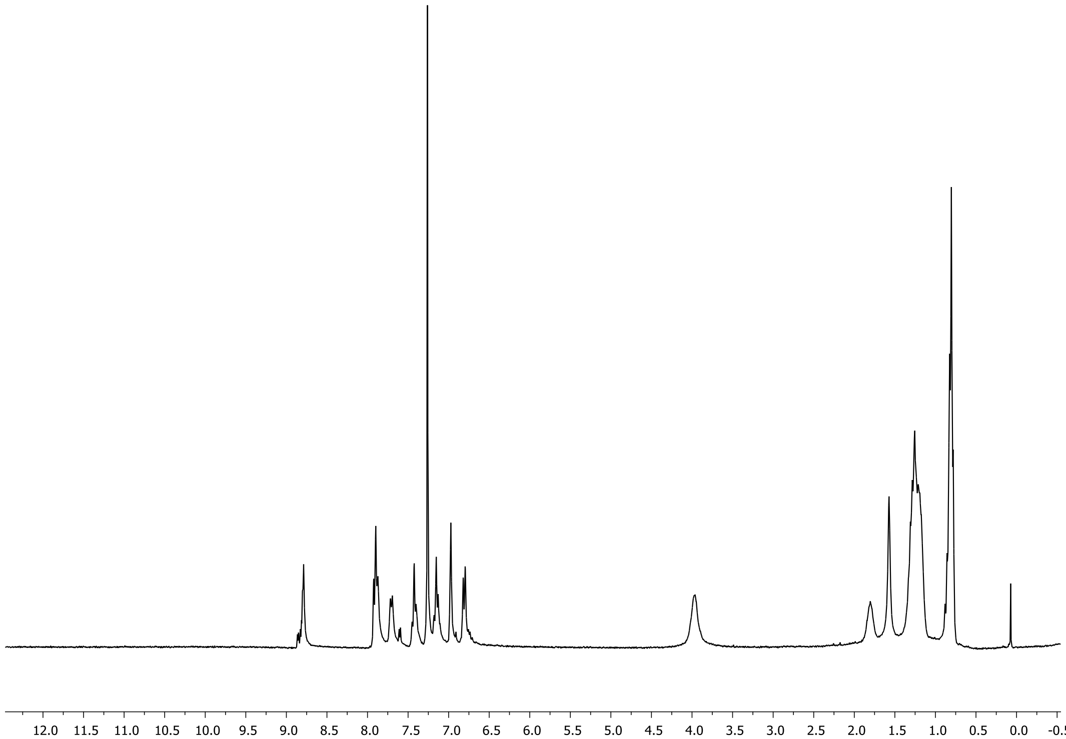

Figure S1. ^1H NMR of **P1** in $CDCl_3$

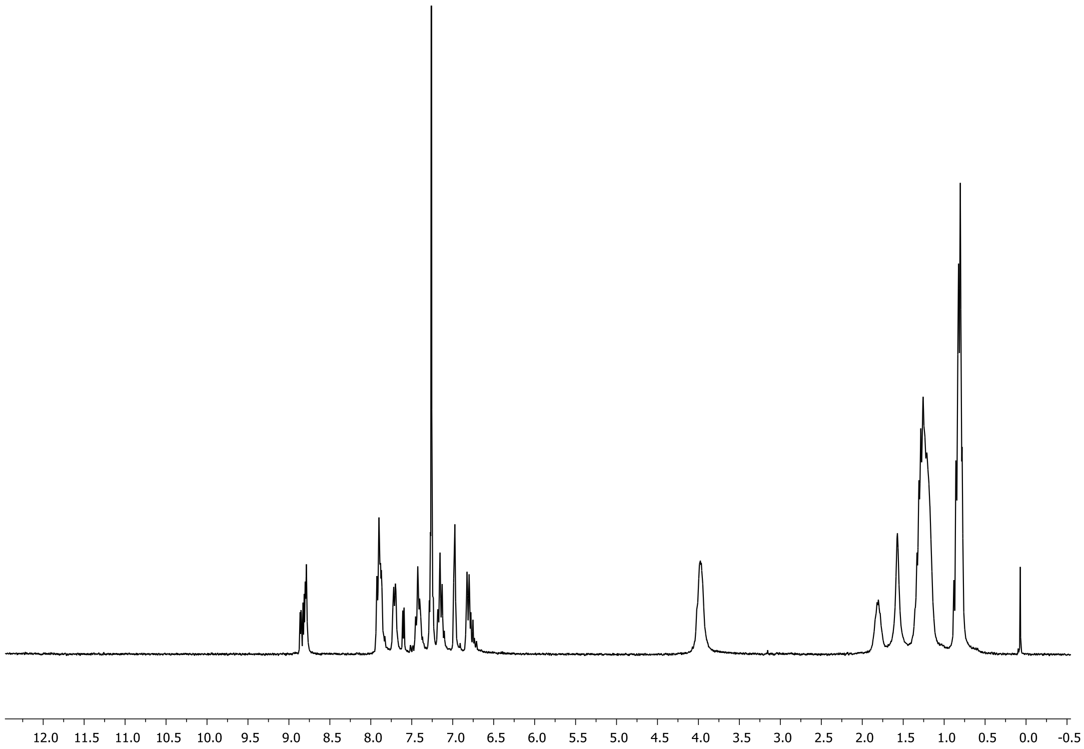

Figure S2. ^1H NMR of **P2** in $CDCl_3$

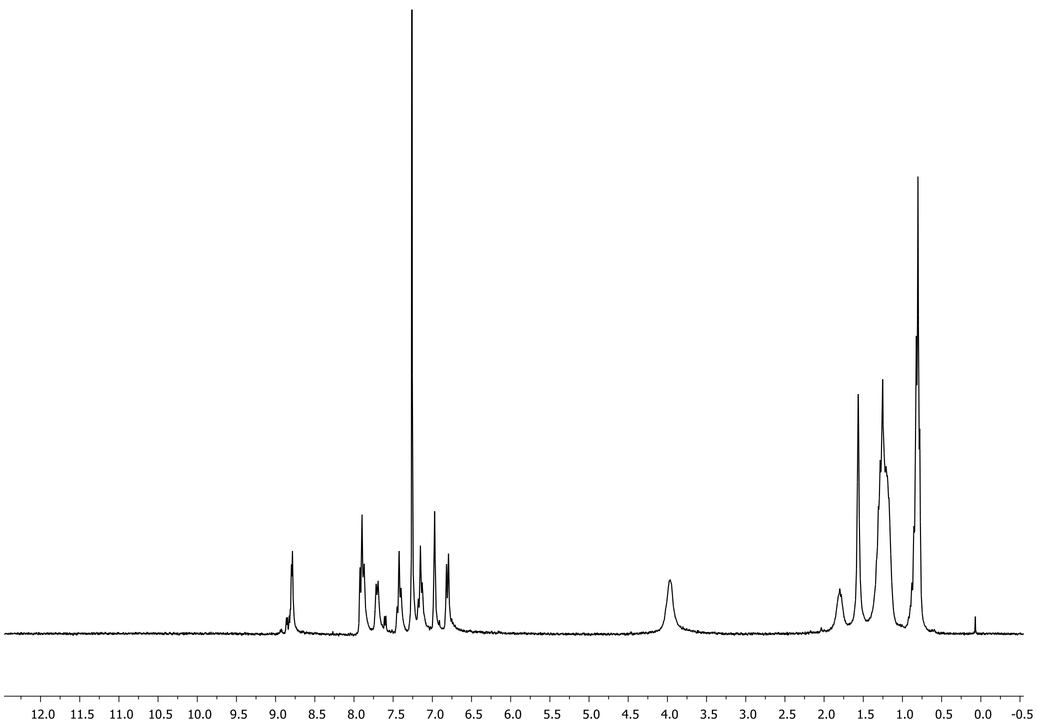

Figure S3. ^{1}H NMR of **P4** in $CDCl_3$

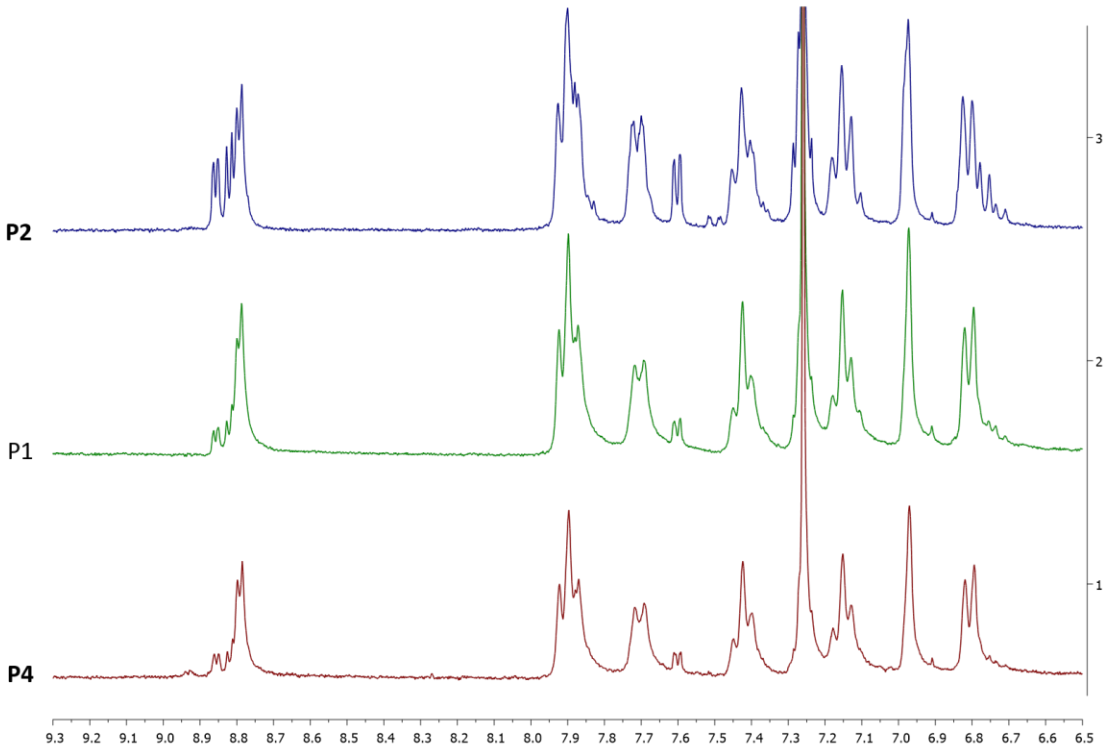

Figure S4. Comparative ^{1}H NMR of **P1** (green); **P2** (blue) and **P4** (red) in $CDCl_3$

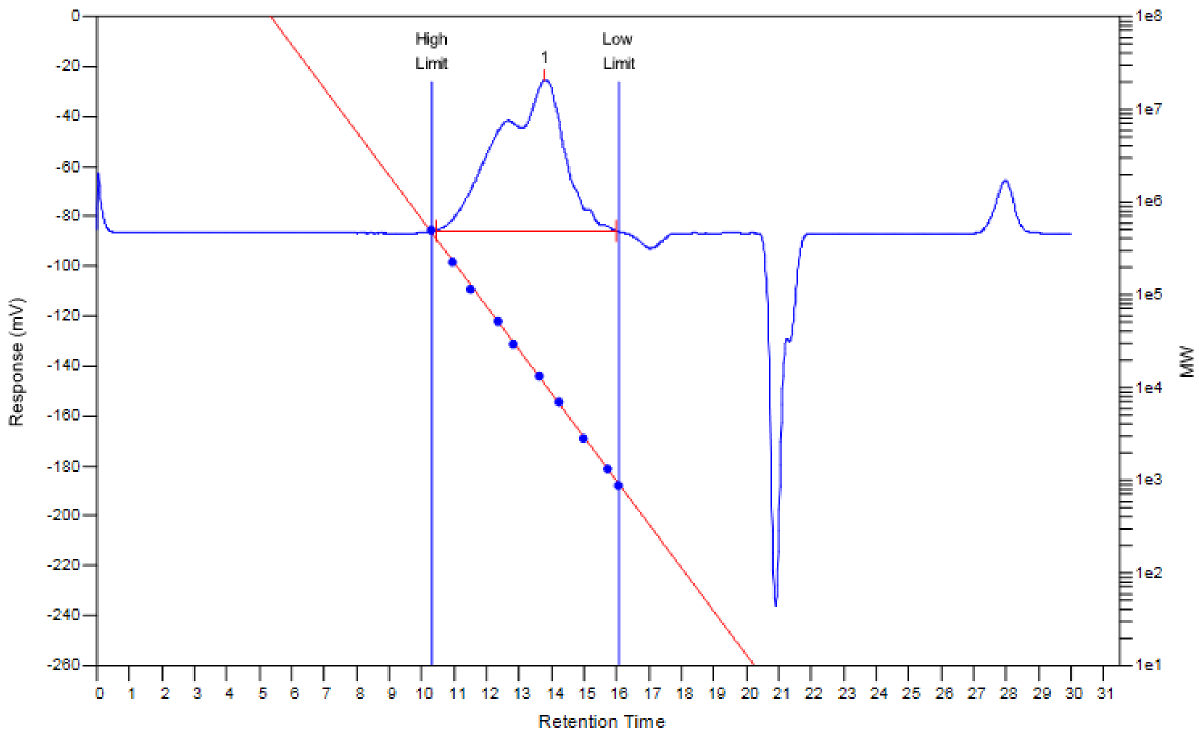

Figure S5. GPC curve obtained for polymer P1

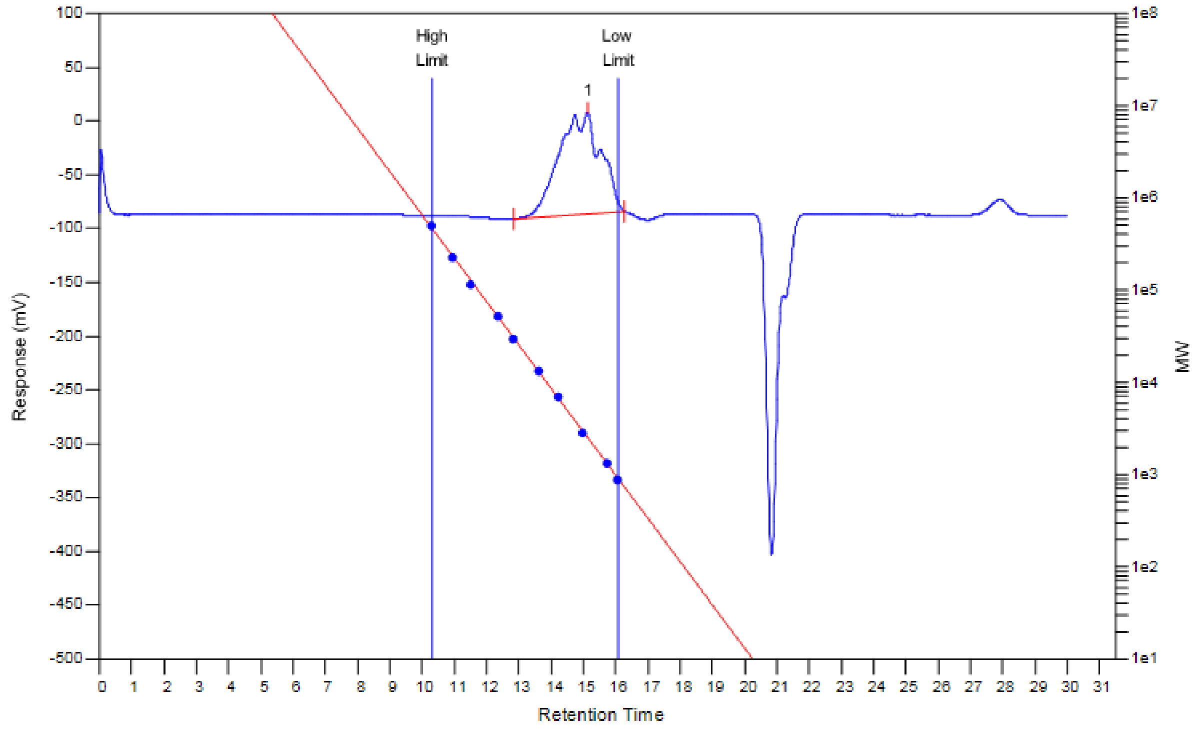

Figure S6. GPC curve obtained for polymer P2

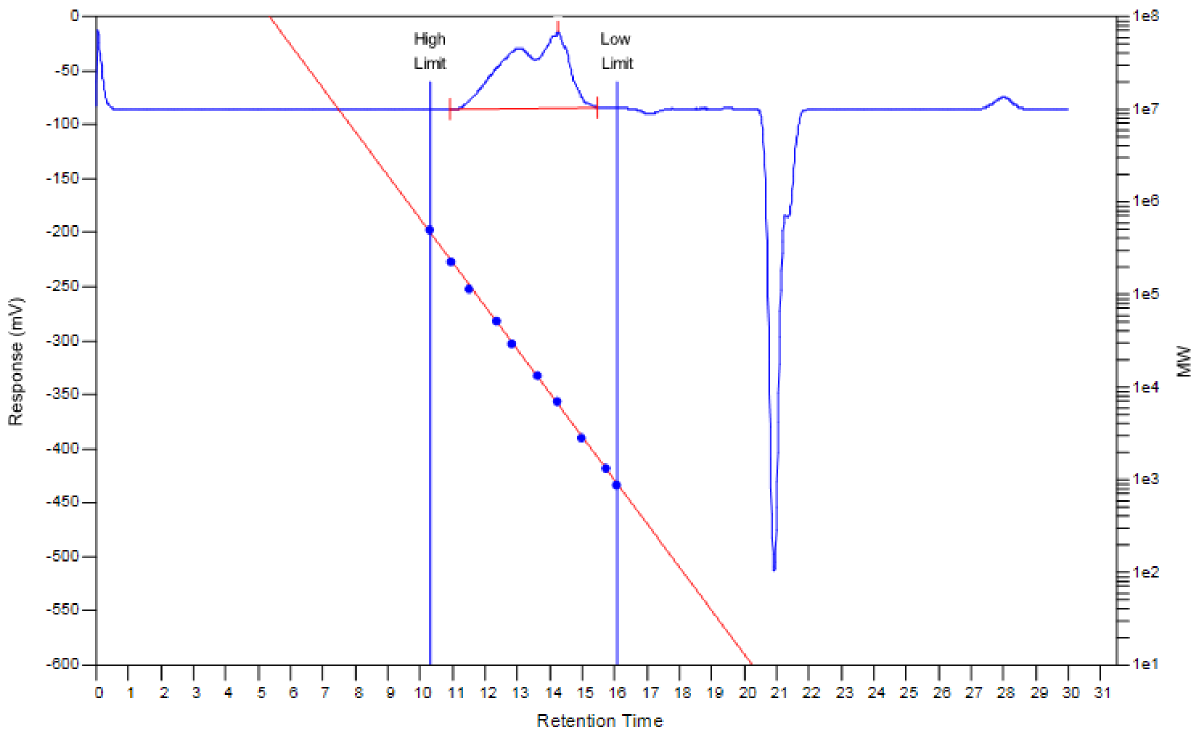

Figure S7. GPC curve obtained for polymer P4

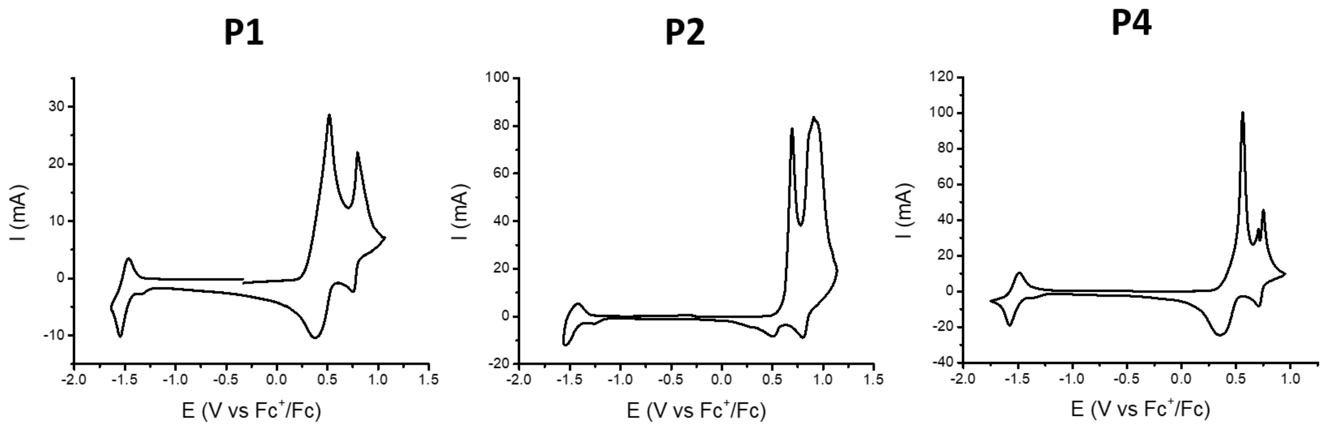

Figure S8. Cyclic voltammogram of **P1**, **P2** and **P4**. Films drop-cast from a $CHCl_3$ solution (5 mg.mL^{-1}) on a Pt WE ; Pt wire CE ; Ag wire pseudo ref ; Fc^+/Fc ref ; 100 mV.s^{-1} in acetonitrile/NBu_4PF_6 (0.1 M)

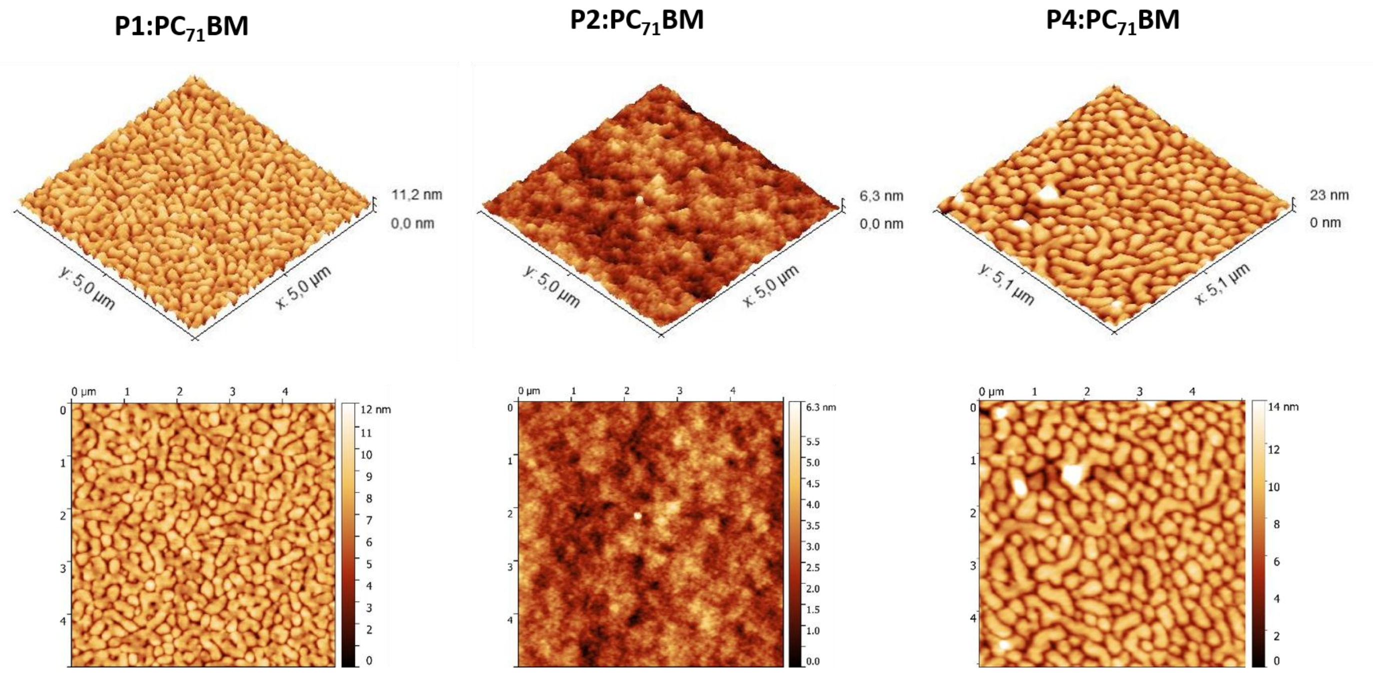

Figure S9. 3D AFM images of **P1**, **P2** and **P4 based** active layers
